# Using online game-based platforms to improve student performance and engagement in histology teaching

**DOI:** 10.1186/s12909-019-1701-0

**Published:** 2019-07-22

**Authors:** Szabolcs Felszeghy, Sanna Pasonen-Seppänen, Ali Koskela, Petteri Nieminen, Kai Härkönen, Kaisa M. A. Paldanius, Sami Gabbouj, Kirsi Ketola, Mikko Hiltunen, Mikael Lundin, Tommi Haapaniemi, Erkko Sointu, Eric B. Bauman, Gregory E. Gilbert, David Morton, Anitta Mahonen

**Affiliations:** 1 Institute of Biomedicine, School of Medicine, Faculty of Health Sciences, University of Eastern Finland, Yliopistonranta 1, 70211 Kuopio, Finland; 2 Institute of Dentistry, Faculty of Health Sciences, University of Eastern Finland, Kuopio, Finland; 3 Institute of Medicine, School of Medicine, Faculty of Health Sciences, University of Eastern Finland, Kuopio, Finland; 4 Fimmic Oy, Helsinki, Finland; 5 Student and Learning Services, University of Eastern Finland, Kuopio, Finland; 6 School of Educational Sciences and Psychology, Philosophical Faculty, University of Eastern Finland, Joensuu, Finland; 7 Clinical Playground, LLC, Madison, WI USA; 8 SigmaStats(r) Consulting, LLC. Charleston, SC USA; 9 Department of Neurobiology and Anatomy, University of Utah School of Medicine, Salt Lake City, UT USA

**Keywords:** Histology education, Medical education, Dental education, Collaborative learning, Game design, Knowledge retention, Whole-slide imaging platform

## Abstract

**Background:**

Human morphology is a critical component of dental and medical graduate training. Innovations in basic science teaching methods are needed to keep up with an ever-changing landscape of technology. The purpose of this study was to investigate whether students in a medical and dental histology course would have better grades if they used gaming software Kahoot® and whether gamification effects on learning and enjoyment.

**Methods:**

In an effort to both evoke students’ interest and expand their skill retention, an online competition using Kahoot® was implemented for first-year students in 2018 (*n* = 215) at the University of Eastern Finland. Additionally, closed (160/215) or open-ended (41/215) feedback questions were collected and analyzed.

**Results:**

The Kahoot® gamification program was successful and resulted in learning gains. The overall participant satisfaction using Kahoot® was high, with students (124/160) indicating that gamification increased their motivation to learn. The gaming approach seemed to enable the students to overcome individual difficulties (139/160) and to set up collaboration (107/160); furthermore, gamification promoted interest (109/160), and the respondents found the immediate feedback from senior professionals to be positive (146/160). In the open-ended survey, the students (23/41) viewed collaborative team- and gamification-based learning positively.

**Conclusion:**

This study lends support to the use of gamification in the teaching of histology and may provide a foundation for designing a gamification-integrated curriculum across healthcare disciplines.

## Background

Students often struggle to appreciate the long-term relevance of histology in understanding the complexity of tissue organization, function and pathological processes [1, 2]. Histology educators also face the daunting task of teaching a large volume of content in a very limited time [3, 4]. Evidence suggests that student-focused approaches improve learning and academic performance compared with more traditional educator-centered strategies [5]. Therefore, educators are interested to implement active learning techniques including gamification to enhance students’ interest in histology and help them to appreciate its clinical relevance [6]. Gamification refers to the use or adoption of game mechanics, techniques and game theory outside the context of traditional game activities or industry into education [7].

Histology education at the University of Eastern Finland (UEF) underwent a crucial curriculum reform in 2016. Student-centered learning and a whole-slide imaging platform were introduced. In practice, students studied in self-guided, slide imaging platform-powered laboratory sessions supervised by a group of professionals. The whole-slide imaging platform-based activities performed by the students were designed to teach histology concepts for each topic of the course. Students followed their syllabus and worked freely. By using the cloud-based histology platform, multiple students could view the same high-quality interactive histological images, and students used this platform on large touch-sensitive screens or on their personal mobile devices. Students could employ the “rotate”, “zoom in/out” and “annotated mode” options to highlight relevant structures, as reported previously by Felszeghy et al. [8]. The supervisors’ role was to support the students, answer questions, and facilitate learning. Student-to-student and student-to-teacher discussions enabled the laboratory sessions to be more productive. The results of our earlier study assessing the new approach in 2017 indicated mastery of the whole-slide imaging platform and positive student attitudes [8].

Studies suggest that students are more likely to remain engaged in an educational activity if technology is involved. Web-based programs, mobile applications and virtual patient simulations are just a few examples of platforms that can incorporate “gamification” [9–13].

**Fig. 1:**
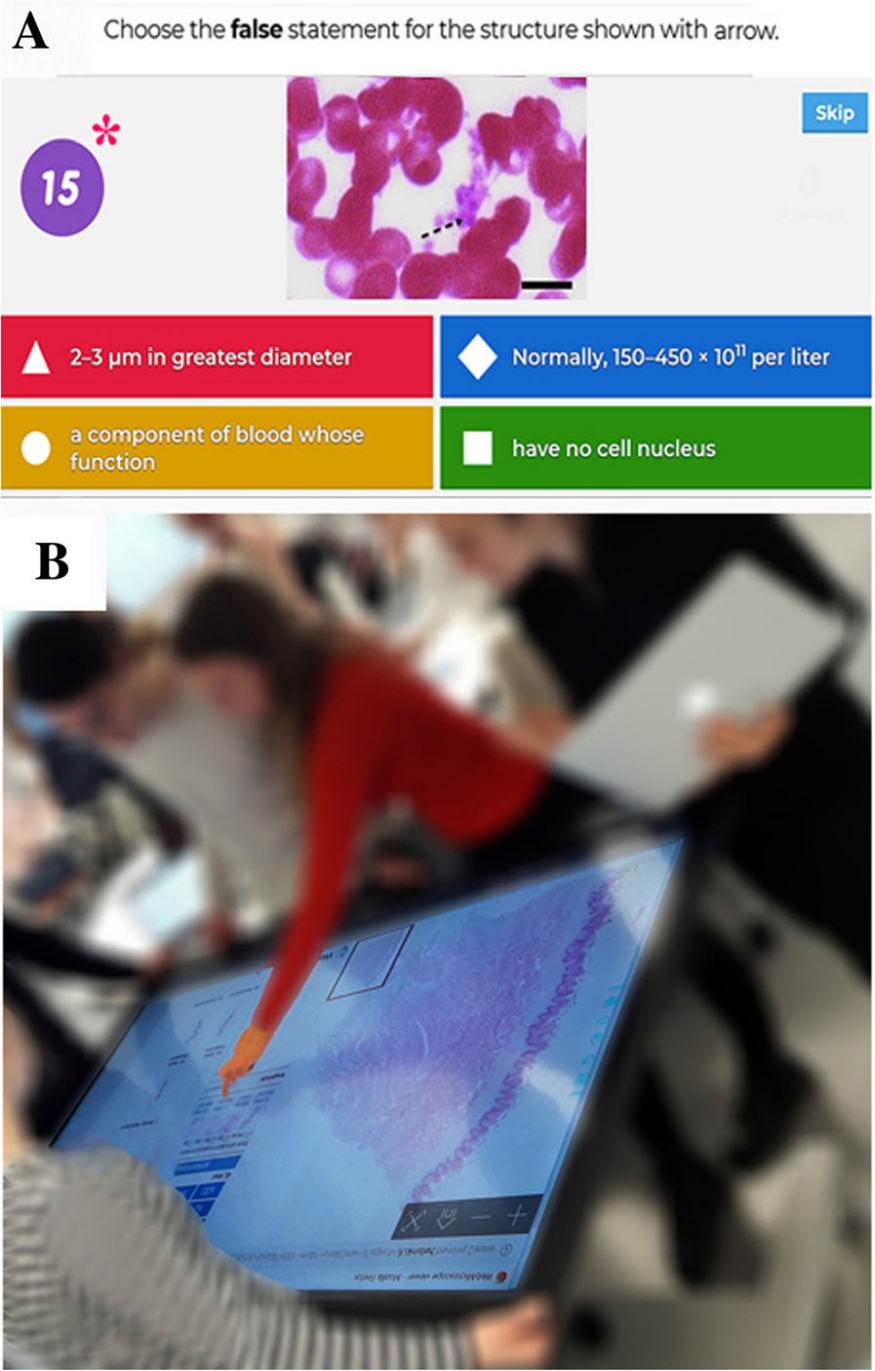
Screenshot of a question and the digital learning scenario. **a** High-quality histological images were used with list of alternative answers to test morphological knowledge as well as correlation with function. Players answered the questions with their own mobile devices, while the questions and the time allocated to answering (red asterisk) were displayed on large shared screens. **b** The rooms for teaching sessions have been set up to move histology education into digital scenarios to help students’ active exchange of histology knowledge, as big touchscreens offered possibilities for teamwork in both years analyzed

One example of a gamified learning environment is Kahoot®, a game-based learning platform freely available on the internet. Kahoot® enables professors to create trivia quizzes in any language and on any device. The quizzes can be utilized on any device and in any location that has cell signal or internet. Kahoot® questions can be utilized in a live, class setting in two ways: 1) Questions are projected on a large screen and each student answers the questions on their mobile device. 2) Students view the questions on their own mobile device and submit the answers. The Kahoot® environment provides time limits and scoring to create a competitive environment. Kahoot® was launched by the Norwegian University of Technology and Science (NTNU) in 2013 (Make Learning Awesome, n.d.). To date, this platform has mainly been actively used as a technology-based tool to facilitate knowledge transfer in business settings, as well as in elementary and middle schools, but it is not yet widely utilized in higher education [14–16]. The major benefit of Kahoot® relative to some other online formative assessment applications (for example, Learning Catalytics™ /Pearson Education Corp., Upper Saddle River, NJ/ Rinaldi et al. [17]) is its ability to display high-quality images or videos with great graphical resolution (Fig. 1).

The literature suggests that gamification increases student engagement and learning. As such, we investigated whether students in a medical and dental histology course using Kahoot® would have increased participation, increased exam performance and better grades than students not using Kahoot®. To our knowledge, this is the first time the use of an online game-based application has been reported in histology teaching with potential ramifications to other subjects within medical and dental curricula.

### Theoretical framework

Bauman’s layered-learning model was chosen as a theoretical framework for this study This model describes a matrix for scaffolding traditional didactic presentation of course materials with multimedia educational technology [18–20]. The model presupposes that traditional didactic learning techniques, such as knowledge transfer through scholarly reading and interaction with faculty and staff, still have relevance in the modern classroom. However, the model leverages contemporary educational technology to scaffold the transfer of knowledge to the learners in a situated and multimodal approach.

Bauman’s layered-learning model does not replace traditional reading assignments with technology but rather provides an approach to learning that increases access to content and avoids the pitfalls of privileging information. By making content available through digital media, simulation can take place anytime, anyplace to enhance the learning experiences. Learners can leverage an array of traditional resources, faculty-led classroom experiences, books (print or digital), and multimedia games or simulations to provide as-needed or just-in-time learning to meet course objectives. Within the context of the Bauman’s model, the role of the faculty shifts from the position of lecturer (“sage on the stage”) to a guide (determines, how best to convey knowledge by leveraging digitally enhanced learning tools and techniques) [21, 22].

## Methods

### Ethical issues

All students were informed that their participation in the research project was voluntary although students were unaware of the specific hypothesis and research question. This study followed the guidelines of the Finnish National Board on Research Integrity and no approval was needed according to the Committee on Health Research Ethics at UEF [23, 24].

### Study design, setting, and participants

The study was conducted in 2018 between March and May using first year medical and dentistry students at University of Eastern Finland (UEF), a public university in Finland. The project employed a quasi-experimental design with five different interventions. The histology course was worth 4.0 credits consisting of 28 lectures and 10 laboratory sessions (2-h per session) and was shared by both the medical and dental students. There was no difference between the histology offered to dental students and that taken by medical students. The course covered the microscopic principles of the human body, from the organization of its cells through major tissues, organs and organ systems. Cloud-based digital histology slides from a variety of human samples were available to students using the whole-slide imaging platform (Aiforia, Fimmic Oy, Helsinki, Finland). This platform enabled virtual slides to be viewed and manipulated by students on large touch-sensitive screens (142.8 cm × 80.4 cm) (SMART Technologies, Huizhou, Korea). The instructor’s helped students build their baseline understanding of the histology they were viewing. In both years analyzed, the educator(s) shared the histology course material (lecture slides, syllabus, recommended books, etc.) with students via UEF’s web-based Moodle platform (software designer: Martin Dougiamas, Perth, Australia).

### Intervention

In 2018 all aspects of the medical and dental histology course were identical to the previous year with the exception of introducing the web-based gamification platform of Kahoot®. Each histology lab session was supervised by experts in histology and digital techniques. Using the syllabus as a reference the students self-guided their learning using the whole-slide imaging platform (Aiforia, Fimmic Oy, Helsinki, Finland). Students received the instructors’ assistance when necessary. Therefore, the histology course in 2018 was similar to 2017 as reported by Felszeghy at al [8] with the exception of introducing Kahoot® in the form of multiple-choice quizzes. To determine the most effective method to employ Kahoot®, the 2018 cohort of first-year medical and dental students were divided randomly into five even sized groups: Group 1 (G1) received their Kahoot® quiz at the beginning of the teaching session (TS) as individual players, Group 2 (G2) received their quiz team-based at the beginning of TS, Group 3 (G3) had the Kahoot® questionnaire introduced at the end of the TS in the individual format, Group 4 (G4) played Kahoot® team-based at the end of the TS, and Group 5 (G5) played Kahoot® game twice, at the beginning and at the end of the TS, as a team. All groups received the same Kahoot® quiz questions. However, between games the order of the questions were randomly changed.

### Outcome

All participants were provided the same quiz questions, which addressed the different topics (nervous tissue, muscle tissue, blood and blood vessels, gastrointestinal tract, and male/female reproductive system). The online Kahoot® quizzes delivered throughout the course had a 15-min time limit. The dependent variable was the histology final exam performance of the 2017 and 2018 cohorts analyzed separately and together.

### The demographic of the study and GPA

The 2017 cohort consisted of 203 students and the 2018 cohort consisted of 215 students. The demographics of medical and dentistry students of UEF shown in Table 1.

**Table 1:**
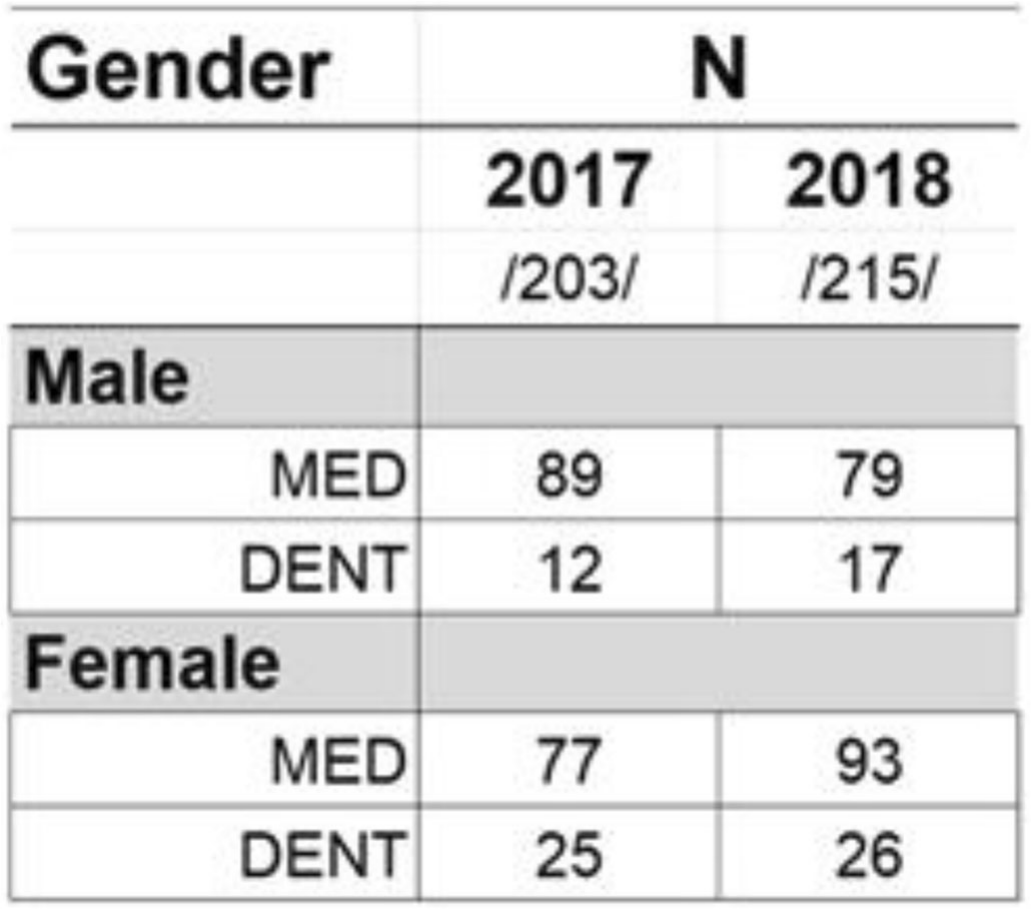
Demographics of Medical (MED) and Dental (DENT) students of University of Eastern Finland. The table displays the sex distribution in medical (MED) and dental (DENT) histology courses at University of Eastern Finland

Among male medical and dental students cumulative grade point (maximum grade:5 and the minimum passing grade:1 and the % of maximum points required for a passing grade is 50%) averages (GPAs) were uniformly lower in 2017 than 2018 cohorts (medical: 3.41 ± 0.51 vs. 3.47 ± 0.39 and dental: 3.20 ± 1.17 vs. 3.22 ± 1.19, respectively). However, the changes between cumulative GPAs were not statistically significant (*p* = 0.710 and *p* = 0.980, respectively). Female medical students had slightly lower cumulative GPAs in 2018 (3.60 ± 1.19 vs. 3.70 ± 1.09). The difference in GPAs for female medical or dental students between 2017 and 2018 was not statistically significant (*p* = 0.529 and *p* = 0.947, respectively)). The GPAs of male medical students increased from 3.06 ± 1.06 to 3.30 ± 1.26 (*p* = 0.140) after implementation of Kahoot®. A similar pattern was also detected among male dental students (2.92 ± 1.00 vs. 3.25 ± 0.87, *p* = 0.463).

### Surveys

Educators conducted meetings to peer-review the quiz questions and surveys prior to circulation to study participants. Content was reviewed by three senior anatomists and by the 2018 histology course coordinator for medical and dental students at UEF. Minor content adjustment was implemented and increased user-friendliness as an online survey was established. The students were asked to voluntarily and anonymously participate in the online surveys in 2018, administered at the end of the histology course. Answers were collected either by Kahoot® using a four-point Likert scale (1 = never, 2 = sometimes, 3 = often, 4 = always) [25] or by Moodle system (open-ended anonymous written feedback). The response rate was approximately 74% (*n* = 160/215) for Kahoot® powered survey, and 75% of the respondents were medical students. Gender distribution was representative of the teaching session as a whole. A descriptive twelve-item questionnaire was developed and administered to participants regarding the merits and impact of using a platform for gamification during basic histology education and to assess the participants’ own preparedness and experiences. Additionally, students were given the opportunity to give open-ended comments on what they liked and disliked in the course with regards to learning and Kahoot® gamification. Nineteen percent (*n* = 41/215) of students offered written feedback. Only information necessary for the monitoring, evaluation, and planning the future directions of histology curriculum was collected. Breakdown analysis for the open-ended questions was applied to analyze these open-ended written data. The full Finnish language version of the data used and/or analyzed during the current study are available from the corresponding author on reasonable request.

### Analysis

Analyses were performed with the SPSS statistical package, v. 23, (IBM Corp., Armonk, NY), and normalized data are presented as the mean ± standard deviation (± SD). One-way analysis of variance (ANOVA) followed by Tukey’s honestly significant difference (HSD) post hoc and Student’s t-test test was conducted for the Kahoot® percentage test score data was used.

for grade point average comparisons, which were transformed to z-scores (standard scores) in order to make the two cohorts comparable. The homogeneity of the variance in the data across the groups was tested with Levene’s test. Qualitative comments in the optional feedback section were subjected to content analysis, whereby themes were identified, and the frequency of occurrence of the themes was determined. Internal consistency of Kahoot® collected survey questions was assessed using Cronbach’s Alpha analysis and those results are presented in the text. Odds ratios (OR) were computed to predict the probability of changes in academic performances. The first specification of the model included histology education in 2018. The reference category was from the year 2017, when no gamification was applied.

## Results

Significantly higher percentages of correct test scores were obtained when students completed the tests in either the beginning of the histology teaching sessions in team mode (G2 69%) vs. individual player mode (G1 58%; *p* < 0.05) or at the end as a team (G4: 87%) vs. individual player mode (G3 82%; p < 0.05). The team-based outcome was enhanced when the students played the online quiz twice, which had a positive effect on the score ratios (G5 90%; p < 0.05). Finally, and interestingly, Ninety-three percent of the total student population participated in the voluntary Kahoot® quizzes during weekly laboratory sessions.

### Questionnaire results

**Fig. 2:**
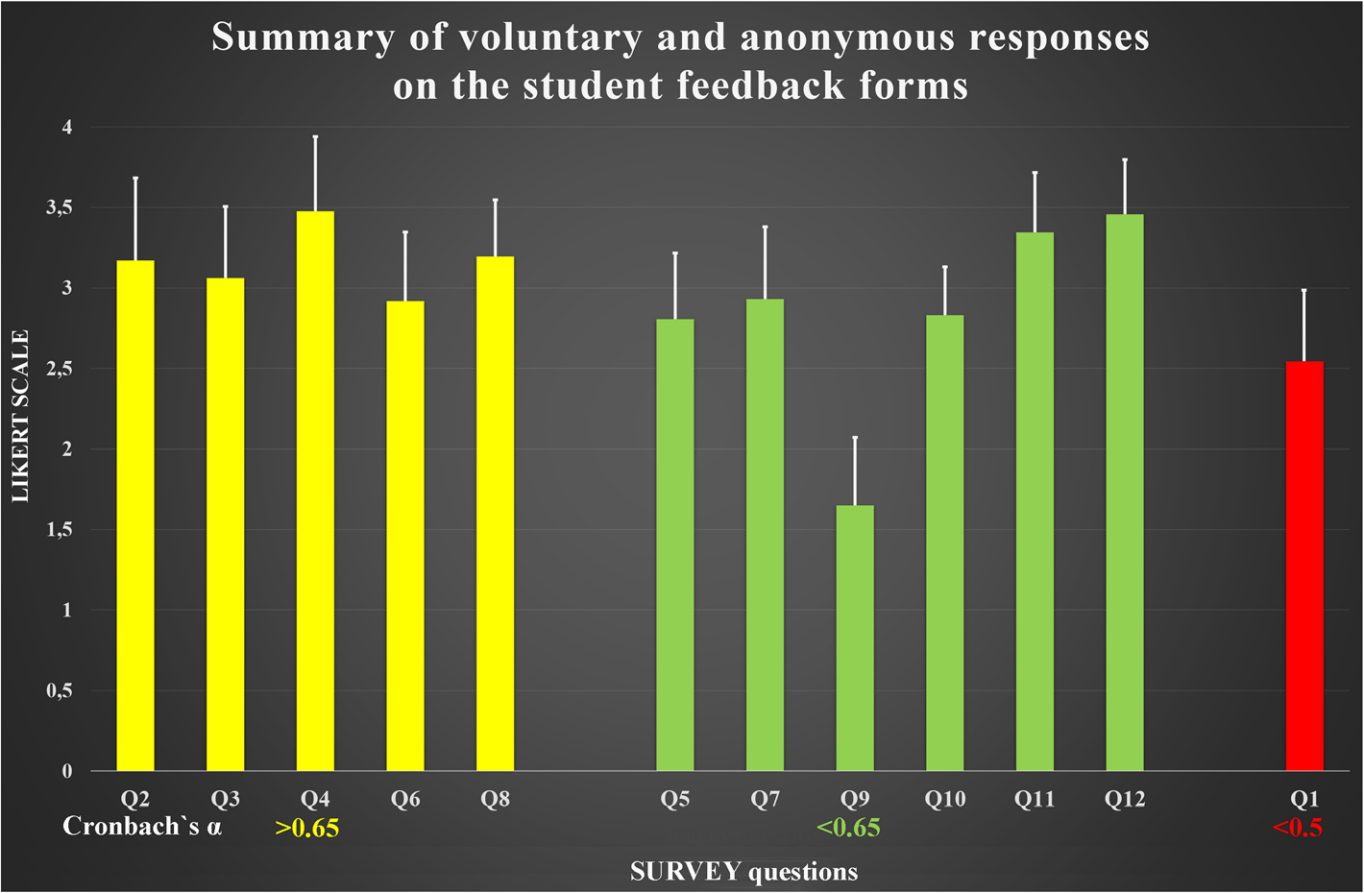
Summary of voluntary and anonymous responses on the student feedback forms**.** The survey represented a snapshot of a single educational event with a relatively large number (160) of attendees. Each person answered one of four levels of agreement or disagreement with the questions raised. Each panel of the plot shows a breakdown of the respondents into categories defined by the criteria listed below in the figure. The results of the survey indicated that the participants’ self-confidence improved with the introduction of gamification, and the students reported that the Kahoot® platform facilitated their ability to learn the course material in a more informal scenario. There was a positive association between the Kahoot® platform and the standardized mastery of the course material, as most of the students who used the platform felt that they had learned the material more comprehensively. Answers were collected by Kahoot® using a four-point Likert scale (1 = never, 2 = sometimes, 3 = often, 4 = always). Results are expressed as means, SD. Cronbach’s alpha coefficients obtained from analysis of questionnaires completed by medical and dental students of University of Eastern Finland was acceptable level coefficient of reliability for question number as follows: yellow bars (Alpha> 0.65): reasonable; green bars: satisfactory (Alpha < 0.65); red bar: unacceptable (Alpha < 0.5). *Abbreviations:* ***Q1:*** *Did you read, printed materials related to topic during the course before each session?* ***Q2:*** *Gamification is an effective method for learning the basics of Histology.* ***Q3:*** *Gamification motivates me to learn more about morphology.* ***Q4:*** *The ability to collaborate with teachers during laboratory session* via *discussion of the game results was important.* ***Q5:*** *Kahoot enhances my understanding on the subjects* ***Q6:*** *Gamification helps to retain my knowledge* ***Q7:*** *The discussions during online game sessions improved my understanding of my skills.* ***Q8:*** *Gamification is an effective method to correct my misconception on the content* ***Q9:*** *I am feeling nervous if I play an online game during laboratory session* ***Q10:*** *The Kahoot is a better platform than other e-learning platforms* ***Q11:*** *I am feeling relaxed if I play an online game during laboratory session.* ***Q12:*** *I’m more engaged with feedback through online gamification*

No missing data imputation strategies were used, thus the final sample size included 160 respondents. The questions listed in Fig. 2 will be referred to as Q1, Q2, etc. according to their sequence number in the questionnaire, to simplify further reading of the text. More than 74% of participants expressed satisfaction with this method of histology education. Internal consistency was assessed using Cronbach’s Alpha which was 0.436 for all twelve survey questions. Elimination process was used to group the questions according to their consistency, i.e. how closely related a set of questions are as a group. Elimination of Q1 resulted satisfactory Cronbach’s alpha level (0.542). Further elimination of Q5, Q7, Q9, Q10, Q11 and Q12 elevated the consistency of remaining questions to a reasonable level (Cronbach’s alpha > 0.650). Therefore, Q2, Q3, Q4, Q6 and Q8 can be considered as reliable group of questions. Eighty-three percent of students reported that this platform created a risk-free anonymous environment in which students could practice without fear of judgment or critical errors while accurately learning and reviewing the concepts of histology. Over 80% of the students reported Kahoot® exercises may have helped set a more relaxed atmosphere for discussions and that the students enjoyed themselves more and were less reluctant to learn the lessons.

Survey results also revealed that students appreciated communication and interactions with their instructors. Approximately 80% of the students employed this option regularly during histology teaching sessions. The participants identified the use of the whole-slide imaging platform and Kahoot® as the particular aspects of the learning environment that they liked the most. Moreover, over 80% of the participants relished the opportunity to obtain constructive feedback from their supervisors in a friendly atmosphere.

In response to queries about gamification in general, the students replied that the Kahoot® system was a useful vehicle enabling them to interact with histology content, either on their own or in a group, and that the Kahoot®-powered assessment model was an effective approach for asking questions about even difficult aspects of histology. It seemed to create an atmosphere of trust, without judgment. In fact, the key feature encountered by students was the ability to feel at ease and to be able to relate to each other, as summarized in Table 2.

**Table 2:**
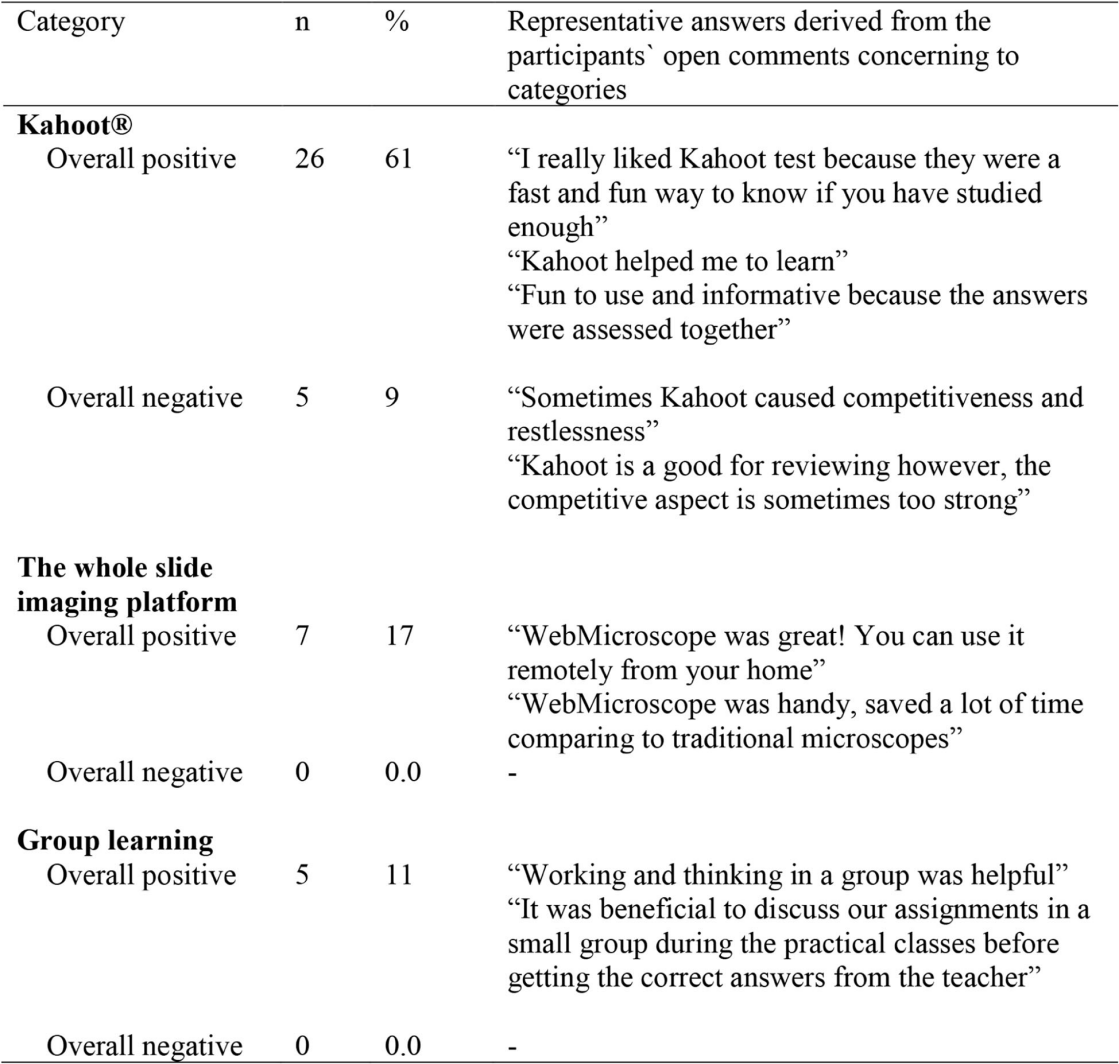
Main themes identified from open-ended questions with regards to learning (What promoted or hindered your learning?)

Narrative feedback was obtained by the students via Moodle, as open-ended questionnaire. Forty-one students out of 215 completed the open-ended survey. Gamification was highlighted by students as positive factor influencing the learning atmosphere. Student comments indicated that gamification created a positive educational experience resulting in a change of pace relative to regular teaching sessions were fun and provided a different motivation to participate. The team-based and teacher-supervised discussions using the whole slide imaging platform appear to be positively evaluated by students

**Fig. 3:**
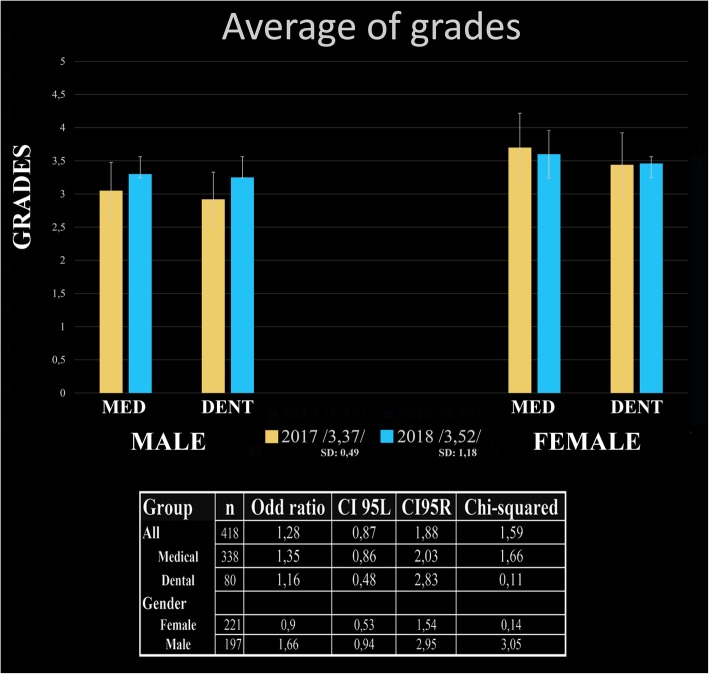
The exam grades earned in 2017 and in 2018 by groups and gender. Displays the exam score averages of different populations of students. A total of 418 students took the written examinations; 203 did so in 2017, and 215 participated in 2018. Introducing gamification into the histology curriculum for the medical and dental population caused a dramatic shift in the distribution frequencies of excellent grades according to odds. The student of 2018 academic year who received gamification based histology education, increased in odds were recorded as compared with students from 2017

Analyses of player attrition, knowledge retention, and final examination performance, for example, make it possible only to describe associations between variables and not causality. Notably, as this study represented standard educational practices, it was not possible to include an actual control group. However, students from 2017 did provide a baseline reference. Based on the analysis of survey data on gamification and on the significantly higher imaging platform accessing activity seen in 2018, we compared the written performances of students between 2017 and 2018. Although the proportion of higher grades increased in all populations in favor of the 2018 scenario, the average scores did not change in a statistically significant manner (Student’s t-test) Fig. 3.

## Discussion

This pilot study explored the effects of online gamification on learning and enjoyment during a medical and dental histology course at UEF. The data from this study suggest some positive benefits of incorporating games into a course, since students felt that the additional competition aspect of the histology course added supplementary educational value, thus confirming a there could be practical implications of gamification at the university level. The population of players who benefited from gamification showed that Kahoot®-mediated teaching sessions strengthened the students’ positive attitudes toward histology teaching sessions in 2018 relative to 2017. Furthermore, our investigation showed that students preferred group or team-based games to individual gaming. When Kahoot® was played as a team, the students were more relaxed according to their self-assessment. This finding is particularly noteworthy, as the repeated introduction and monitoring of new information via quiz assessment was more beneficial to the sustained retention of information, which has also been suggested by other studies [26, 27]. With gamification, although students adopted the habit of starting to perform a task upon entering the histology teaching sessions, the percentage of errors in student answers was lower in practice with the repeated quiz than when a single questionnaire was given at only one time point in a particular histology session. When a large amount of complex information is provided in a short period of time (i.e., during a 100-min histology lab session), the lesson material may be partially or entirely forgotten by some students [28]. The results indicate that the amount of information learned during teaching session may be more easily recalled by students if they are quizzed on smaller amounts of information but more frequently, as was the case the gamified intervention leveraged in this study. We also found that students preferred the team-based approach to evaluation of knowledge transfer. In other words, completing quizzes as a group rather than individually. The impact and development of team interaction in the context of team-based reasoning skills is important for students future academic and professional careers [8, 29, 30, 31].

The overall student performance for the different gamification scenarios was rather high. One possible explanation is related to our choice of question formats, which included multiple choice, true/false, matching, and mapping identification questions. Each of the Kahoot® questions were reviewed for content validity, which concerns, primarily, the adequacy with which the test items adequately and representatively sample the content area to be measured. As reported previously by Cortright and colleagues, student disinterest is a critical factor in weak performance in higher education, which was certainly not the case in our study, as it is worth noting that acceptable number of students (92/215) used printed material regularly to prepare for weekly histology sessions [32] (Fig. 2).

**Fig. 4:**
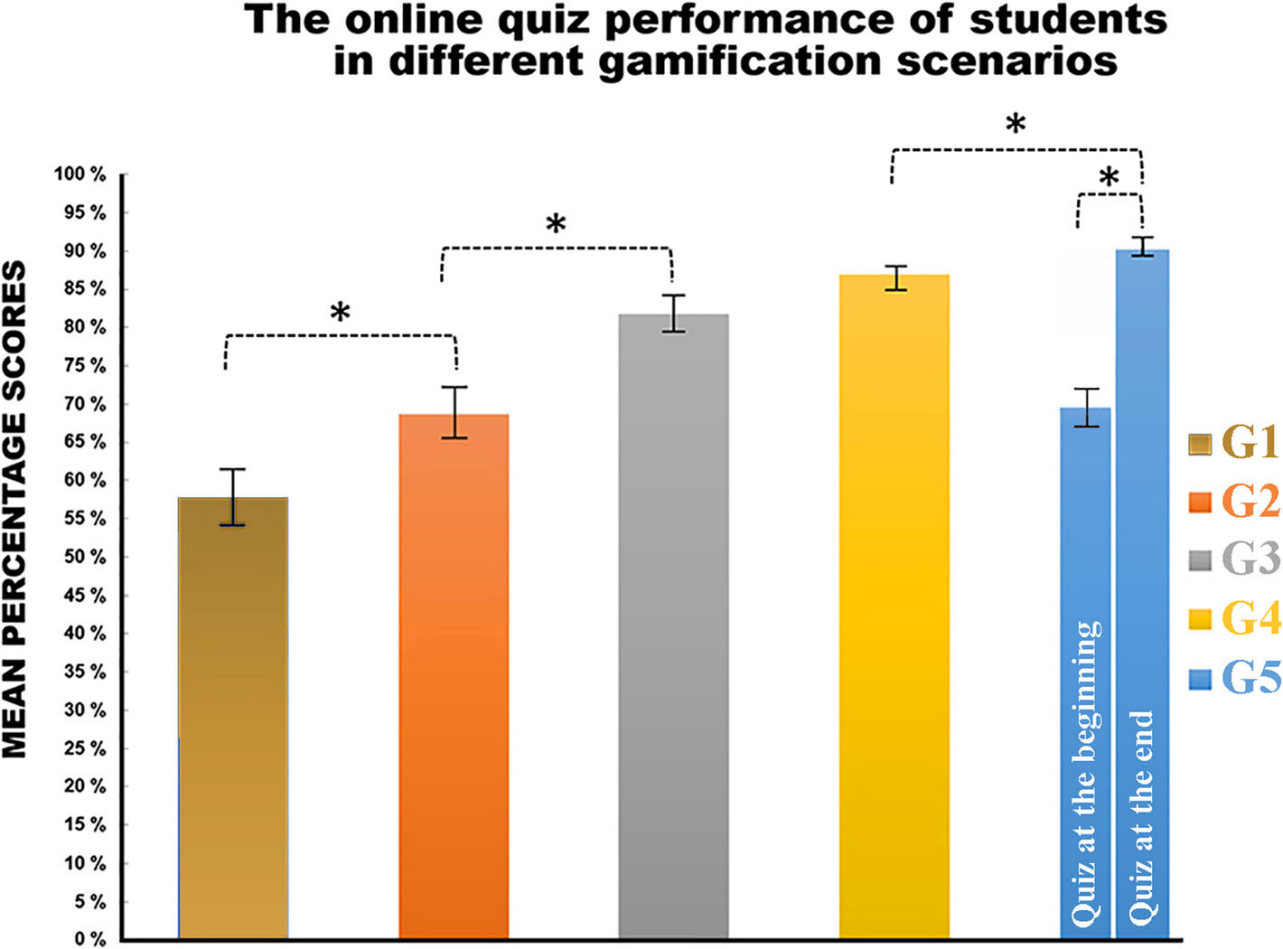
The topic-relevant online quiz performance of students in different gamification scenarios. Experimental groups: online quiz played at the beginning of teaching sessions by an individual player (GI) or a team (G II), quiz played at the end of teaching session by an individual player (G III) or a team (G IV), and quiz played twice, at the beginning and the end of class, by a team (G V). The first-year medical and dental students from the University of Eastern Finland were randomly grouped into even-sized groups. *denotes a significant difference between groups, *p* < 0.05, one-way ANOVA

In this investigation, 63% of students felt that interactive gamification helped increase their knowledge of basic histology. Eighty-five percent of the survey participants reported that they gained a better understanding of histology content. The results indicate that the just-in-time feedback from the Kahoot® software facilitated team-based discussions and encouraged peer-to-peer learning. Our findings suggest that gamification used as part of group active learning approach [33] improved histology education. This is illustrated by higher percentages of correct answers when the intervention took place in team-based mode rather than in individual mode (Fig. 4). Further, our findings are consistent with those of Hrynchak and Batty [34], who reported that gamification enhances the formation of an active learning community, where knowledge is conveniently and easily accessible. Our findings are consistent with previous studies that found that Kahoot® incentivized group learning and problem-solving skills [35]. In addition, the data presented as part of this investigation indicate that team-based gamification supports peer-to-peer and small group learning among students. Timely and just-in-time qualitative and quantitative feedback among team members playing together may engage students in ways that are beneficial to the broad spectrum of students found within in a class or cohort of students and may positively impact their learning outcomes.

The survey also showed that learning readiness after gamification was highly associated with being prepared. In other words, the more prepared the students were for their topic, the more accurately and actively they participated in the Kahoot® discussions. Interestingly, even the less-prepared students were satisfied with how Kahoot® advanced their practical capabilities and formed a new interactive, relaxed communication venue between students and supervisors.

Finally, another interesting aspect of our results was that medical and dental students also preferred traditional written material for the histology course and regularly employed these materials to prepare themselves in advance. The students were probably looking for relevant, focused information from books or other written material available on the Moodle site of UEF. It has also been suggested that students might read more slowly, less accurately and less comprehensively on screens than on paper [36–38]. One possible explanation is that more traditionally minded students might want to keep printed materials for future reference. This third option is indirectly supported by survey answers in which participants highlighted the importance of course-related materials on Moodle for their studies, which were even more important than their own notes developed during different sessions of the histology course.

The data suggest that the Kahoot® platform enhanced learning by enabling students to engage histology topics via gamification and leverage other materials such as video links. In this way, the Kahoot® platform provided a layered learning model that presented multiple types of media to scaffold the curriculum. This approach was consistent with the redesigned histology curriculum, which applied just-in-time formative assessment opportunities for students based on the weekly learning objectives. Further gamification scenarios encouraged a deeper understanding of the materials.

### Limitations of the study

Due to the mandatory nature of histology course at UEF and the middle scale of the entering students, the first limitation of the study was the inability to have a proper control group that participated in the study but not the competition component.

Second, this study was conducted at a single institution with a discrete population, medical and dental students. Future studies should explore the influence of student characteristics on academic performance in other settings. Third, comparing these results to those of future cohorts of students and evaluating the role of gamification in knowledge retention represents important next steps. Fourth, self-assessment may have more relevance in regard to learner attitude and confidence. However, self-assessment by students as it relates to knowledge gain is of limited value in terms of program goals and outcomes. Moreover, the Kahoot® platform was not specially developed for histology education, or higher education, rather, Kahoot® offers a generic tool for a wide variety of educational content dissemination. While there was change in the proportions of higher grades after gamification, we cannot exclude the possibility of repeated testing in combination with the gamification quizzes. It is thus plausible that similar improvements could have been attained by using other methods that include repeated testing. However, gamification also offered other positive outcomes including the student’s perceptions of a friendly atmosphere and general satisfaction with the game quiz — also important factors in student motivation and ultimately performance.

Finally, these results suggest that even though gamification seem to be effective in teaching histology in certain contexts, this assumption cannot be generalized without further research.

## Conclusions

As more digital natives enroll in higher education, medical educators need to incorporate frameworks, such as Bauman’s Layered Learning Model, when designing active learning procedures. Our study demonstrated that the introduction of gamification into dental and medical histology course stimulated learning and improved participant satisfaction. The qualitative portion of the study, i.e., the students’ answers to open-ended questions, supported the theoretical framework suggesting that educators can consider integrating new digital technologies into curricula. New technologies, such as the introduction of Kahoot® or similar gamification platforms, could complement the more traditional teaching methods and provide additional scaffolding for student learning.

## Data Availability

The datasets used and/or analysed during the current study are available from the corresponding author on reasonable request.

## Abbreviations

UEF: University of Eastern Finland
